# External irradiation models for intracranial 9L glioma studies

**DOI:** 10.1186/1756-9966-29-142

**Published:** 2010-11-08

**Authors:** Sandrine Vinchon-Petit, Delphine Jarnet, Eric Jadaud, Loïc Feuvret, Emmanuel Garcion, Philippe Menei

**Affiliations:** 1 INSERM, U646, Université d'Angers, Angers, F-49100, France; 2 Department of Radiation Therapy, Centre Paul Papin, Angers, F-49100, France; 3 Department of Radiation Therapy, Hôpital de la Pitié-Salpêtrière, Paris, F-75013, France; 4 Department of Neurosurgery, CHU d'Angers, Angers Cedex 9, F-49933, France

## Abstract

**Purpose:**

Radiotherapy has been shown to be an effective for the treatment human glioma and consists of 30 fractions of 2 Gy each for 6-7 weeks in the tumor volume with margins. However. in preclinical studies, many different radiation schedules are used. The main purpose of this work was to review the relevant literature and to propose an external whole-brain irradiation (WBI) protocol for a rat 9L glioma model.

**Materials and methods:**

9L cells were implanted in the striatum of twenty 344-Fisher rats to induce a brain tumor. On day 8, animals were randomized in two groups: an untreated group and an irradiated group with three fractions of 6 Gy at day 8, 11 and 14. Survival and toxicity were assessed.

**Results:**

Irradiated rats had significantly a longer survival (p = 0.01). No deaths occurred due to the treatment. Toxicities of reduced weight and alopecia were increased during the radiation period but no serious morbidity or mortality was observed. Moreover, abnormalities disappeared the week following the end of the therapeutic schedule.

**Conclusions:**

Delivering 18 Gy in 3 fractions of 6 Gy every 3 days, with mild anaesthesia, is safe, easy to reproduce and allows for standardisation in preclinical studies of different treatment regimens glioma rat model.

## Background

Malignant glioma is the most frequent primary brain tumor. Prognosis is extremely poor with current standards of treatment. Median survival is less than fifteen months with a multimodality treatment of surgery, radiotherapy (RT) and chemotherapy [1]. Temozolomide, a novel alkylating agent, has shown modest activity against recurrent glioma. However, in newly diagnosed patients with glioblastoma, temozolomide in combination with radiotherapy significantly prolongs survival. Radiotherapy represents a significant part of the treatment regimen for malignant glioma [2-4]. To be sufficiently efficacious with acceptable toxicity, RT consists of 30 fractions of 2 Gy each, usually administered Monday-Friday for 6-7 weeks (42 days) in the tumor volume with margins. The schedule is clearly defined and established in clinical practice [5]. Consequently, in preclinical studies evaluating adjuvant therapies, radiation therapy should be included. Previously, we used a fractionated radiation schedule delivering 36 Gy in 9 fractions of 4 Gy to treat C6 tumor bearing-rats [6]. We found that brain radiotherapy for rat 9L-glioma, which is the most common preclinical model used, is not standardized. Moreover, the schedules described in literature are highly heterogeneous (Table 1) [6-13]. To prove a potentially promising effect of a concomitant treatment and to compare different study results, the radiation therapy protocol must be well defined. Following a review of the literature, the aim of this study is to propose a brain irradiation protocol for rats that is closer to clinical practice, safe for small animals and easy to reproduce in the study of concomitant treatments for glioma.

**Table 1:** Studies using radiation therapy rat model in combination with anticancer therapeutic agents

*Studies*	*Target*	*Tumor Cell line*	*Total dose*	*Number of fractions*	*Survival*
Roullin VG (6)	HB	C6	36 Gy	9	Complete response : 8%
Graf MR (7)	WB	T9	15 Gy	1	35 days (median)
Kimler BF (8)	WB	9L	20 Gy	1	S
			30 Gy	5	S
Kimler BF (9)	WB	9L	40-70 Gy	10-20	S
Kimler BF (10)	WB	9L	16 Gy	1	38.5 days (mean)
Kimler BF (11)	WB	9L	16 Gy	1	S
			24 Gy	1	S
			32 Gy	1	S
			40 Gy	1	S
Lamproglou I (12)	WB	-	30 Gy	10	-
Olson JJ (13)	WB	9L	30 Gy	1	29.7 days (mean)

WB: Whole brain/HB: Hemibrain/S: Significant

NB: Lamproglou worked on normal rat brains.

## Methods

All experiments have been conducted under good experimental practices. All animal handling was carried out according to the European Community regulations and French Ministry of Agriculture regulations.

### Animals

20 females Fischer-344 rats were used for this study (Charles River, Cleon, France). Rats were ten weeks-old, and weighed 150 to 200 grams. They were housed in groups of 4 in cages according to the standards of the directives of the European Union. Animal handling was conducted by the animal facility of the Faculty of Medicine of Angers, approved according to French law.

### Tumor model

Rat 9L-glioma cells (European Collection of Concealment Culture, n° 94110705, Salisbury, U.K.) were cultured in "DMEM" medium ("Dulbecco's Modified Eagle's Medium", Biowhittaker, Verviers, Belgium) with 10% foetal calf serum (FBS, Biowhittaker) and a mixture of antibiotics: penicillin (100 UI/ml), streptomycin (0.1 mg/ml) and amphothericin B (25 μg/ml) (ABS, Sigma, Saint Quentin Fallavier, France). Cells were maintained in a balanced wet atmosphere (37°C and CO_2 _5%).

Animals were anesthetized with an intraperitoneal injection of 0.75-1.5 ml/kg of a solution containing 2/3 ketamine (100 mg/ml) (Clorketam^®^, Vétoquinol, Lure, France) and 1/3 xylazine (20 mg/ml) (Rompun^®^, Bayer, Puteaux, France). Rats were placed in a small-animal stereotaxic frame (Kopf Instruments, Phymep, France). After shaving and disinfection of the skin, a sagittal incision of 2 cm was made to expose the skull, followed by a burr hole 0.5 mm anterior and 3 mm lateral from the bregma using a small drill.

Following trypsinisation (trypsin/EDTA (Sigma)) and resuspension in "EMEM" ("Eagle's Minimum Essential Medium", Biowhittaker), 10 μl of 10^3 ^9L-cells in suspension were implanted 5 mm deep in the right striatum (according to the Paxinos atlas) using a 10 μl -26G Hamilton syringe (Harvard Apparatus, Ulis, France). After waiting 5 minutes, the needle was removed and the wound was sutured with absorbable surgical thread.

Rats bearing 9L tumor were randomized to either the "untreated" group (group A) or the group irradiated by a whole-brain irradiation (WBI) to a total dose of 18 Gy (group B). The radiotherapy started on day 8 after the tumor cell implantation when the tumor size was 10-15 μl [14].

### Radiotherapy protocol

**Figure 1:**
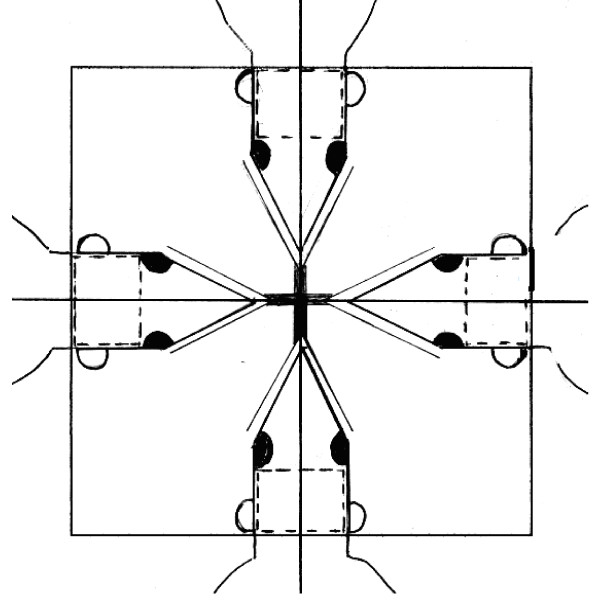
**Radiation therapy position**. An equivalent tissue of 1.5 cm was laid on the rat head in order to improve the dose distribution in brain.

Rats were irradiated using a 6-MV linear accelerator (Saturn 41 type, Varian Medical Systems, Salt Lake City, USA), under mild anaesthesia by isoflurane (4.5% during 2 minutes then 2% for the treatment) + O_2 _3 L/min. Oxygen masks were connected and four rats were placed in a reproducible way, in a prone position on the linac couch with laser alignment. The WBI was delivered by one photon beam (6 MV-energy, DSP 100 and 4 Gy/min). The radiation field was 15 × 15 cm at source-axis distance of 100 cm. The isocenter was in the midline of the brain and the posterior limit of the field corresponded to the line passing by the posterior part of the 2 ears (Figure 1).

**Figure 2:**
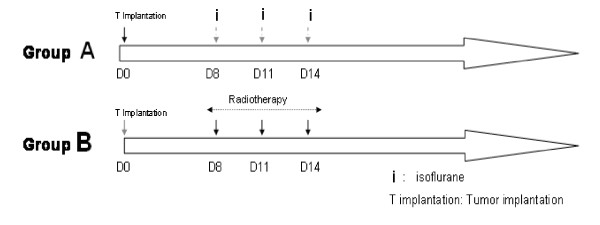
**Therapeutic schedule**.

A 15-mm thickness of equivalent tissue was laid on the rat's head in order to improve dose distribution to the brain. The dose distribution was defined by the Radiation Therapy department. Eighteen Gy, given in 3 fractions of 6 Gy were delivered over 7 days in the isocenter corresponding to the tumor (Figure 2). The brain was covered by the 95%-isodose. The irradiation was only started in the absence of wound healing problems (abscess, haematoma...) and if rat's general state allowed it. After irradiation, animals were replaced in their cage. Control rats were also anesthetized according to the same schedule as the group B animals.

### Animal observation

Rats were examined daily and staged for activity and well-being according to a classification developed in our animal facility (data not published) (Table 2). Toxicities were noted. Rats were weighed weekly. Rats too weak to feed and to stand (corresponding to stage 2) were sacrificed (atmosphere saturated with CO_2_). The day of euthanasia was recorded and used in the survival analysis. All brains were removed and macroscopically examined when possible. It was noted if a tumor was found.

**Table 2:** Rats staging (data not published)

	Stage 5	Stage 4	Stage 3	Stage 2	Stage 1
Motility	Normal	Normal	+ but not spontaneous	Reduced	No

Stature	Normal	Stooped +	Stooped ++	Stooped +++	Dying

Piloerection	No	+/-	+++	+++	+++

Eyes	sharp	Redness+	Redness ++	Eye secretions	closed

### Statistics

Survival was calculated from the day of the tumor implantation and presented as median and mean ± SE (Standard Error). Increase of life span (ILS) was calculated as follows: (Mean Survival Max - Mean Survival Min)/Mean Survival Min × 100. A Student t-test was performed to compare mean survival in the two groups, using SPSS^® ^software and tests were considered as significant with p values < 0.05. Any rat surviving longer than 120 days was defined as a 'long survivor'. The Kaplan-Meier method was used to plot animal survival. Animals that died during anesthesia were not included in the survival analysis.

## Results

### Efficacy of the brain irradiation

**Figure 3:**
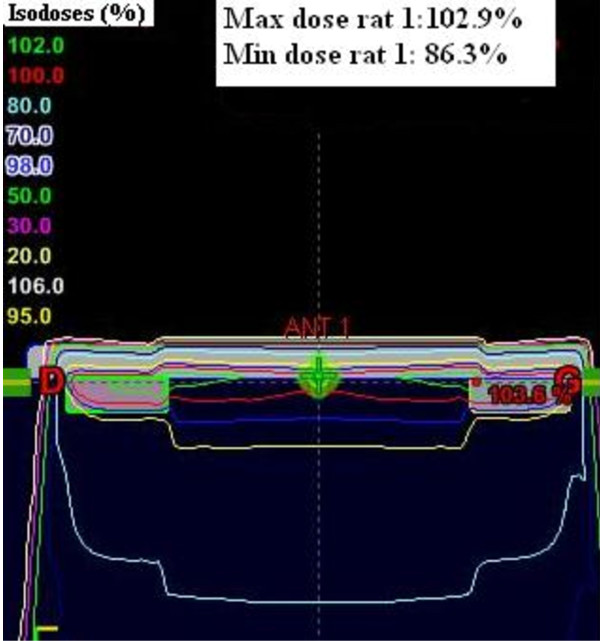
**Dose distribution in the whole rat brain**.

**Figure 4:**
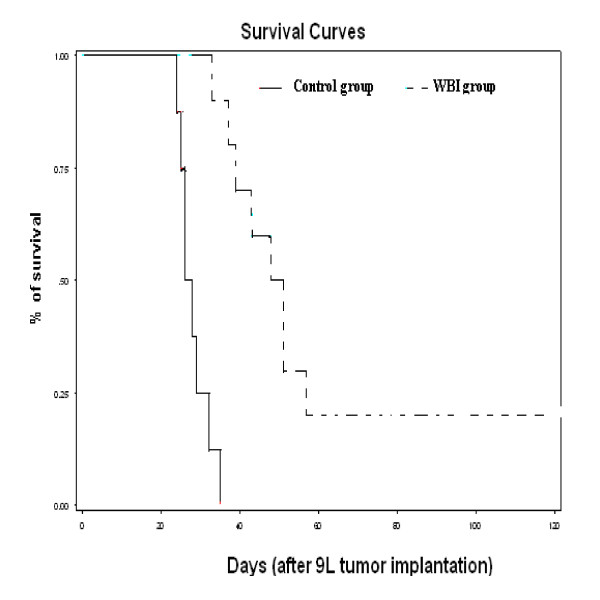
**Survival curves depending of each group of treatment**. Survival times (days) after tumor implantation have been plotted for "untreated animals" (Group A) and "WBI (3 fractions of 6 Gy)" animals (group B).

The dosimetry planning is reported in figure 3. The 95%-isodose curve covered all the brain and 95% of the volume received 95% of the total dose. In the group A, two animals died during anaesthesia induction, before the tumor cells implantation. The brain was analyzed macroscopically in 12 animals (six in group A and six in group B). Deterioration of the brain in other animals, due to oedema, prevented analysis. For the 12 animals, a large tumor was observed in their right striatum. By day 35, all rats in group A died. Mean survival of this untreated group was 28.1 days ± 1.3. For group B, mean survival was 59.9 days ± 8.2 (Table 3). The rate of long survivors in this group was 20% (2/10 rats). The macroscopic examination of their brain was normal, with no sign of tumor or injection trail; therefore we did not perform a microscopic analysis. Rats treated with WBI showed an increased mean survival span (ILS) of 113% when compared to controls. Survival time was significantly longer compared to the control group (p = 0.01) (Figure 4).

**Table 3:** Descriptive and statistical data from the survival study depending on groups of treatment

*GROUPS*	*Median of survival (days)*	*Mean time of survival (days) ± SE*	*Mean ILS (%)*	*Long term survivors*	*Maximal time of survival (days)*
**Group A « untreated » (n = 8)**	27	28.1 ± 1.3	-	0	35

**Group B « WBI » (n = 10)**	49.5	59.9 ± 8.2	113	2	120

WBI: Whole brain irradiation

ILS: increase in lifetime span

### Schedule toxicity

**Figure 5:**
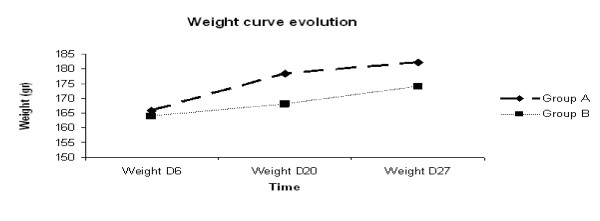
**Evolution of the weight median depending on time of observation according to the group**.

No rat, in any group, developed evident behavioural anomalies until approximately four days preceding death. Rats of the 2 groups were either sacrificed at stage 2 to avoid suffering or died spontaneously during the night (n = 8). The others twelve rats were found dead in the morning. There were no issues with wound healing following the procedure. All rats in group B developed incomplete and reversible (WHO grade II) alopecia at the surgical site during radiation therapy. Animals recovered by 21 days following the last day of irradiation. During the radiation therapy (d8-d14), the general behaviour was maintained, with no feeding trouble although the weight increase was slower than observed for rats in group A. For group A, weight gain was typical for twelve week-old rats. The mean increase in weight for the "untreated" group A was 7.69% between d8 and d20 versus 2.47% for the WBI group (figure 5). This difference was significant (*p *= 0.01). In a previous study (14), mean time of survival of the untreated group was 27.5 days; loss of weight would have been noted for a significant number of rats due to neurological deterioration related to the tumor progression. So, for group A, values of the weight increase after day 20 resulted from an extrapolation starting from the weight increase noted during the first 14 days. Weight gain was no longer significantly different one week after the end of radiation therapy (day 21) (p = 0.25) with an increase of weight estimated at 3.79% for group A and 6% for the group B (figure 5). No other clinical abnormalities due to irradiation were observed.

## Discussion

Even though single-fraction irradiation was reported to be well tolerated in the literature, we decided to use a fractionated radiotherapy protocol to irradiate rats, as this is closer to clinical practice and more adapted for a preclinical study, especially with daily concomitant chemotherapy as defined by Stupp for human gliomas [1]. In the literature, from 5 to 20 fractions have been delivered in the preclinical studies we reviewed (Table 1) [[6,8,9] and [12]]. One potential limitation of fractionated radiotherapy for small animals is the reproducibility of positioning. In these small animal models, rats have to be anesthetized, especially if one hemi-brain irradiation is required. However, most drugs used for anaesthesia have effects on blood brain pressure, which is already high when a brain tumor grows, or are known to be radioprotective for the normal brain parenchyma. Ketamine, which is commonly used for anaesthesia of rodents, induces a general increase in cerebral blood flow at anaesthetic concentrations [15]. Some authors reported that pentobarbital protects against radiation-induced damage to normal rat brain. Even though there is no conclusive evidence for either radioprotection or significant improvement of radiotherapeutic efficacy, in 9L rat brain tumor model pentobarbital could potentially induce the selective protection of normal brain [11,13]. In our model, anaesthesia with isoflurane is easy to use every three days, is well tolerated by rats with a complete and immediate recovery after irradiation and does not interfere with normal or brain tumor cells.

**Figure 6:**
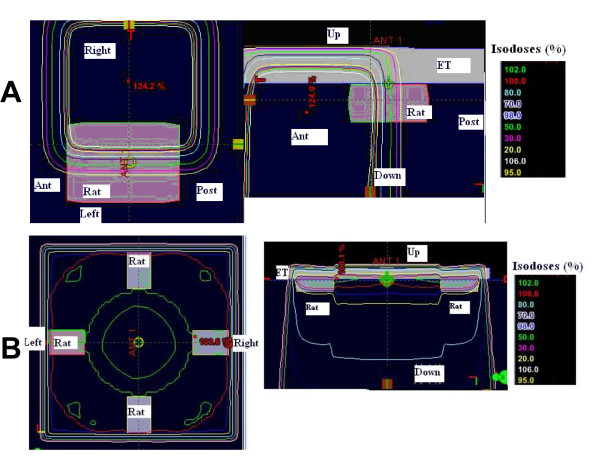
**Dose distribution in one hemibrain (A) and in the whole rat brain (B)**.

**Figure 7:**
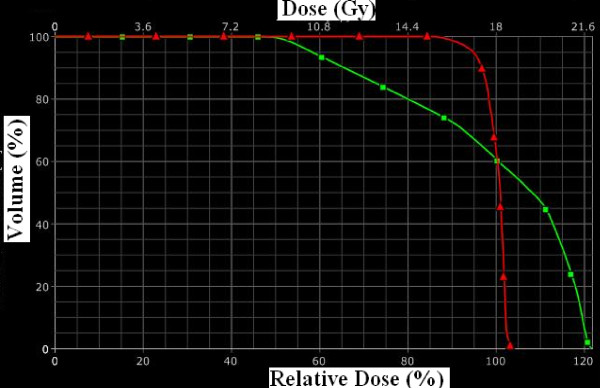
**Histogram-Dose Volume according to the treatment received**. Green: hemibrain irradiation. Red: whole brain irradiation.

Some investigators use Plexiglas stereotactic frames for rat positioning and treat just one hemi-brain. Previously, in our laboratory, we used a fractionated radiotherapy in one hemi-brain [6]. We found that the volume of interest is better covered when the whole brain is treated, as opposed to hemi-brain irradiation, due to the small size of a rat brain (figure 6). The Dose Volume histogram (DVH) obtained for these two treatment modalities are represented in figure 7.

The optimal dose per fraction to treat a rat brain glioma is not well defined. Our protocol was selected based on the linear-quadratic formula with α/β of 10 for the tumor and α/β of 3 for the normal tissue. The effective biological dose for the normal tissue is 32 Gy and 27 Gy for the tumor. These doses correspond to the dose received in clinical practice for a whole brain irradiation. 9L cells are classified as a radioresistant cell line especially compared to other rodent glioma cell lines [16]. Bencokova described a surviving fraction at 2 Gy (SF2) of 71.9% for 9L cells against 53.0 and 41.4% for C6 and F98 cell lines respectively [16]. According to this, the dose to deliver by fraction must be higher than 2 Gy. The dose per fraction in literature ranges from 2 to 40 Gy (Table 1). For Kimler, the survival improvement was limited by the development of normal tissue toxicity at high doses [11]. Kim observed that 35 Gy produced severe optic neuropathy [17]. In his study, he tested a single high dose of radiation (ranging from 20 to 45 Gy) with radiosurgery in a limited volume. Previously, we investigated a radiation therapy schema in 3 fractionated doses of 6 Gy a week *in vitro *on 9L cell lines without and with concomitant chemotherapy [18]. The results showed that cell death was most important as the number of fractions increased from 1 to 3 and the increase was higher for the schemas associated with chemotherapy. For all the conditions tested, the greatest cell death was obtained after the first fraction (60-75% cell death), and was slightly reduced after the second and the third fraction. On the other hand, the most important observation was the synergistic effect between chemotherapy (CT) and RT which was most evident after the third fraction, as cell death increased from 5.3% to 38.2% for the cells treated with RT alone versus CT + RT, respectively. After the third fraction, the cell percentage still alive was mainly due to the radioresistance mechanism described above. Taking these findings into consideration, with the aim of finding a treatment protocol that is efficacious but not toxic, we chose to deliver 3 fractions of 6 Gy in our model of rat glioma. With this schedule we noted a mild and transitory toxicity which was quickly reversible after treatment.

Two rats in the WBI group lived more than 120 days. They were sacrificed and their brain was removed; there was no sign of tumor. It is not possible to determine whether there was a technical problem during the tumor cells implantation or if the animals achieved a complete response after irradiation.

There is a paucity of experimental data in literature on rat radiobiology. Different energy sources are used. Some groups work with a dedicated irradiator for small animals in their laboratory. This type of irradiator uses ^137^Cesium or ^60^Cobalt source and delivers gamma-rays [[9,19,20] and [21]]. As Lamproglou, even though his work was on normal brain [12], we decided to treat our rats with linear accelerator used in clinical practice. Animal irradiation may be difficult to manage because of the limited availability of accelerators but the main advantage is to deliver the same energy type as in clinical practice. There are other advantages of using a nonradioactive x-ray-producing irradiator such as avoiding the increasing number of radioprotection controls as well as the potential source hazard, disposal and replacement; nonetheless the expected efficacy is the same whatever the radiation source chosen.

This work does not answer the crucial question of optimal therapeutic regimen as it was conducted before our studies into the efficacy of local chemotherapy concomitant to radiation therapy in 9L glioma [22]. Another study confirms the reproducibility of the model as we obtained the same improvement in survival in the radiation group compared to the untreated group [18]. Therefore, this radiation therapy protocol has the potential to induce strong tumor debulking and facilitate concomitant chemotherapy treatment.

## Conclusion

Many models of radiation therapy for rat glioma are available, with different schedules. We describe a reproducible paradigm of fractionated radiotherapy for rat bearing a brain tumor, which reflects clinical practice, with a good compromise between feasibility and adaptation to chemotherapy radiosensitization studies.

## Competing interests

The authors declare that they have no competing interests.

## Authors' contributions

SVP carried out the studies and drafted the manuscript. DJ carried out the irradiations. EJ and LF participated in the drafting. EG and PM participated in the design of the study. All authors read and approved the final manuscript.

## References

[B1] Stupp R Mason WP van den Bent MJ Weller M Fisher B Taphoorn MJ Belanger K Brandes AA Marosi C Bogdahn U Curschmann J Janzer RC Ludwin SK Gorlia T Allgeier A Lacombe D Cairncross JG Eisenhauer E Mirimanoff RO European Organisation for Research and Treatment of Cancer Brain Tumor and Radiotherapy Groups; National Cancer Institute of Canada Clinical Trials Group Radiotherapy plus concomitant and adjuvant temozolomide for glioblastoma N Engl J Med 2005 352 10 987 96 10.1056/NEJMoa043330 15758009

[B2] Kristiansen K Hagen S Kollevold T Combined modality therapy of operated astrocytomas grade III and IV. Confirmation of the value of postoperative irradiation and lack of potentiation of bleomycin on survival time: a prospective multicenter trial of the Scandinavian Glioblastoma Study Group Cancer 1981 47 4 649 52 10.1002/1097-0142(19810215)47:4<649::AID-CNCR2820470405>3.0.CO;2-W 6164465

[B3] Laperriere N Zuraw L Cairncross G Cancer Care Ontario Practice Guidelines Initiative Neuro-Oncology Disease Site Group: Radiotherapy for newly diagnosed malignant glioma in adults: a systematic review Radiother Oncol 2002 64 3 259 73 10.1016/S0167-8140(02)00078-6 12242114

[B4] Cairncross G Berkey B Shaw E Phase III trial of chemotherapy plus radiotherapy compared with radiotherapy alone for pure and mixed anaplastic oligodendroglioma: Intergroup Radiation Therapy Oncology Group Trial 9402 J Clin Oncol 2006 24 18 2707 14 10.1200/JCO.2005.04.3414 16782910

[B5] Kantor G Laprie A Huchet A Loiseau H Dejean C Mazeron JJ Radiation therapy for glial tumors: Technical aspects and clinical indications Cancer Radiother 2008 12 6-7 687 94 18926759 10.1016/j.canrad.2008.09.004

[B6] Roullin VG Mege M Lemaire L Cueyssac JP Venier-Julienne MC Menei P Gamelin E Benoit JP Influence of 5-fluorouracil-loaded microsphere formulation on efficient rat glioma radiosensitization Pharm Res 2004 21 9 1558 63 10.1023/B:PHAM.0000041448.22771.48 15497679

[B7] Graf MR Prins RM Hawkins WT Merchant RE Irradiated tumor cell vaccine for treatment of an established glioma. I. Successful treatment with combined radiotherapy and cellular vaccination Cancer Immunol Immunother 2002 51 4 179 89 10.1007/s00262-002-0269-3 12012105 PMC11032860

[B8] Kimler BF Martin DF Evans RG Morantz RA Vats TS Effect of spirogermanium and radiation therapy on the 9L rat brain tumor model NCI Monogr 1988 6 115 8 3352753

[B9] Kimler BF Martin DF Evans RG Morantz RA Vats TS Combination of radiation therapy and intracranial bleomycin in the 9L rat brain tumor model Int J Radiat Oncol Biol Phys 1990 18 5 1115 21 1693363 10.1016/0360-3016(90)90447-r

[B10] Kimler BF Liu C Evans RG Morantz RA Combination of aziridinylbenzoquinone and cis-platinum with radiation therapy in the 9L rat brain tumor model Int J Radiat Oncol Biol Phys 1993 26 3 445 50 8514542 10.1016/0360-3016(93)90962-u

[B11] Kimler BF Liu C Evans RG Morantz RA Effect of pentobarbital on normal brain protection and on the response of 9L rat brain tumor to radiation therapy J Neurosurg 1993 79 4 577 83 10.3171/jns.1993.79.4.0577 8410227

[B12] Lamproglou I Chen QM Boisserie G Mazeron JJ Poisson M Baillet F Le Poncin M Delattre JY Radiation-induced cognitive dysfunction: an experimental model in the old rat Int J Radiat Oncol Biol Phys 1995 31 1 65 70 7995769 10.1016/0360-3016(94)00332-F

[B13] Olson JJ Friedman R Orr K Enhancement of the efficacy of x-irradiation by pentobarbital in a rodent brain-tumor model J Neurosurg 1990 72 5 745 8 10.3171/jns.1990.72.5.0745 2324799

[B14] Vonarbourg A Sapin A Lemaire L Characterization and detection of experimental rat gliomas using magnetic resonance imaging Magma 2004 17 3-6 133 9 10.1007/s10334-004-0049-5 15503254

[B15] Laitio RM Kaisti KK Låangsjö JW Aalto S Salmi E Maksimow A Aantaa R Oikonen V Sipilä H Parkkola R Scheinin H Effects of xenon anesthesia on cerebral blood flow in humans: a positron emission tomography study Anesthesiology 2007 106 6 1128 33 10.1097/01.anes.0000267596.57497.92 17525587

[B16] Bencokova Z Pauron L Devic C Molecular and cellular response of the most extensively used rodent glioma models to radiation and/or cisplatin J Neurooncol 2008 86 13 21 10.1007/s11060-007-9433-0 17611717

[B17] Kim JH Khil MS Kolozsvary A Fractionated radiosurgery for 9L gliosarcoma in the rat brain Int J Radiat Oncol Biol Phys 1999 45 4 1035 40 10571213 10.1016/s0360-3016(99)00273-4

[B18] Allard E Passirani C Jarnet D Petit S Vessières A Jaouen G Benoit J-P Local delivery of ferrociphenol lipid nanocapsules followed by external radiotherapy as a synergistic treatment against intracranial 9L glioma xenograft Pharm Res 2010 27 1 56 64 10.1007/s11095-009-0006-0 19908129

[B19] Kinsella TJ Kinsella MT Hong S Toxicology and pharmacokinetic study of orally administered 5-iodo-2-pyrimidinone-2'deoxyribose (IPdR) × 28 days in Fischer-344 rats: impact on the initial clinical phase I trial design of IPdR-mediated radiosensitization Cancer Chemother Pharmacol 2008 61 2 323 34 10.1007/s00280-007-0518-4 17562042

[B20] Brust D Feden J Farnsworth J Radiosensitization of rat glioma with bromodeoxycytidine and adenovirus expressing herpes simplex virus-thymidine kinase delivered by slow, rate-controlled positive pressure infusion Cancer Gene Ther 2000 7 5 778 88 10.1038/sj.cgt.7700168 10830725

[B21] Yacoub A Hamed H Emdad L MDA-7/IL-24 plus radiation enhance survival in animals with intracranial primary human GBM tumors Cancer Biol Ther 2008 7 6 917 33 10.4161/cbt.7.6.5928 18376144

[B22] Vinchon-Petit S Jarnet D Paillard A Benoit JP Garcion E Menei P In vivo evaluation of intracellular drug-nanocarriers infused into intracranial tumours by convection-enhanced delivery: distribution and radiosensitisation efficacy J Neurooncol 2010 97 2 195 205 Epub 2009 Sep 22 10.1007/s11060-009-0012-4 19768659

